# What inference for two-stage phase II trials?

**DOI:** 10.1186/1471-2288-12-117

**Published:** 2012-08-06

**Authors:** Raphaël Porcher, Kristell Desseaux

**Affiliations:** 1, Univ Paris Diderot, Sorbonne Paris Cité, Unité de Biostatistique et Epidémiologie Clinique, UMR-S717,, Paris, F-75010, France; 2 Département de Biostatistique et Informatique Médicale, Hôpital Saint-Louis, AP-HP, Paris, F-75010, France; 3, INSERM, U717, Paris, F-75010, France

## Abstract

**Background:**

Simon’s two-stage designs are widely used for cancer phase II trials. These methods rely on statistical testing and thus allow controlling the type I and II error rates, while accounting for the interim analysis. Estimation after such trials is however not straightforward, and several different approaches have been proposed.

**Methods:**

Different approaches for point and confidence intervals estimation, as well as computation of *p*-values are reviewed and compared for a range of plausible trials. Cases where the actual number of patients recruited in the trial differs from the preplanned sample size are also considered.

**Results:**

For point estimation, the uniformly minimum variance unbiased estimator (UMVUE) and the bias corrected estimator had better performance than the others when the actual sample size was as planned. For confidence intervals, using a mid-*p* approach yielded coverage probabilities closer to the nominal level as compared to so-called ’exact’ confidence intervals. When the actual sample size differed from the preplanned sample size the UMVUE did not perform worse than an estimator specifically developed for such a situation. Analysis conditional on having proceeded to the second stage required adapted analysis methods, and a uniformly minimum variance conditional estimator (UMVCUE) can be used, which also performs well when the second stage sample size is slightly different from planned.

**Conclusions:**

The use of the UMVUE may be recommended as it exhibited good properties both when the actual number of patients recruited was equal to or differed from the preplanned value. Restricting the analysis in cases where the trial did not stop early for futility may be valuable, and the UMVCUE may be recommended in that case.

## Background

Phase II trials primarily aim at evaluating the activity of a new therapeutic regimen to decide if it warrants further evaluation in a larger-scale phase III trial, where it is usually compared to a standard treatment. The screening purpose of phase II trials implies that they are designed to reject a new therapeutic regimen showing low therapeutic activity. In cancer phase II trials, therapeutic activity is typically defined in terms of tumor shrinkage [1,2], and a patient with tumor shrinkage is referred as a responder. The endpoint of such phase II trials is thus a binary endpoint (responder / nonresponder), and a new anticancer agent with too low a response rate should be excluded from further consideration.

Cancer phase II trials are often designed as multistage trials (two stages being most common) allowing early trial termination in case of a low response rate, in order to avoid giving patients an ineffective treatment and wasting resources. The original idea of such a strategy with early termination was suggested by Gehan [3], and many designs were then proposed ([4-6], among others). Among all available multistage designs, Simon’s design [6] is probably the most commonly used in practice. Conversely, early termination for high efficacy is not as important in the phase II setting. Actually, there are less ethical needs to stop the trial early for an effective agent, and accumulating data on both therapeutic activity and safety is important before setting up a large-scale randomized phase III trial.

As phase II trials primarily lead to the decision to proceed to a next step in the evaluation of the therapeutic regimen or not, their design essentially relies on statistical testing. Cancer phase II trials are therefore designed to control the probabilities to continue with an ineffective regimen or to abandon an effective one (type I and II error rates, respectively). Further analysis, and in particular estimation, is nevertheless useful and usually conducted, especially if the new regimen is selected for further consideration [7,8]. A point estimate of the response rate, a confidence interval and sometimes a *p*-value are then computed at the termination of the trial. In particular, the point and confidence interval estimates are useful to design the future phase III trial, as well as other phase II trials. Owing to the possibility of early termination, the sample response rate, i.e. the maximum likelhood estimator (MLE), is typically biased, which is known as the optional sampling effect. Many approaches have thus been proposed to reduce the bias or the mean squared error (MSE) of estimators in such a setting [7,9-14].

One important point concerning inference in two-stage phase II trials has been somewhat overlooked in the literature. As estimation is most important when the therapeutic regimen has been considered as effective, inference may be more common when the phase II trial proceeded to the second stage as compared to cases where it was stopped for futility at the first stage. Inference may thus be conditional on proceeding to the second stage (as e.g. in [12,13]), or unconditional, over all possible paths as implicitely considered in most other works.

Another issue is the actual total sample size of the trial. Cancer phase II trials are generally of limited sample size, and methods are derived from the ’exact’ binomial distribution of data. However, the actual number of patients recruited in the trial may be different from the planned sample size [11,15]. Inference in a Simon’s design where the sample size has been modified is however not straightforward, even in terms of hypothesis testing. A method has thus been proposed in the case where drop-outs are non-informative so that the interim analysis can always be performed after inclusion of the planned number of patients and the actual second stage sample size does not depend on results observed during the first stage [11]. Although designs where the second stage sample size can be adapted according to the first stage result exist [16,17], this was not considered here.

In this paper, we compare the performance of the different approaches proposed in the literature for inference in a two-stage Simon’s phase II trial. In the next section, we present the different point estimators, confidence intervals and *p*-values proposed in the case where the actual sample size is as planned and in the case where the actual stage 2 sample size of the trial is different from the planned one. Then, results of a numerical study comparing the properties of the different methods in various settings are presented. We conclude with some discussion.

## Methods

### Simon’s design and notations

Let us denote *Π*as the true response rate when given some anticancer agent. Usual methodology of cancer phase II trials consists in testing the null hypothesis *Π*≤*Π*_0_versus *Π*≥*Π*_1_ = *Π*_0_ + *δ*, where *Π*_0_ is the highest probability of response which would indicate that the agent is of no further interest, and *Π*_1_the smallest probability of response indicating that the agent may be promising. Simon [6] considered two-stage designs where no stopping for efficacy is possible after the first stage. Briefly, *n*_1_subjects are accrued during the first stage. If the number of responses observed in the first stage *X*_1_ is lower or equal to a critical value *r*_1_, the trial is stopped for futility. If *X*_1_>*r*_1_, the trial proceeds to a second stage where *n*_2_ additional patients are accrued. Let us denote *X*_2_ the number of responses observed in the *n*_2_second stage patients, *X*_*t*_ = *X*_1_ + *X*_2_and *r*_*t*_ the final critical value. Then if *X*_*t*_≤*r*_*t*_futility is concluded at the end of the trial, whereas efficacy is concluded if *X*_*t*_>*r*_*t*_. Given (*Π*_0_*Π*_1_) many such two-stage designs may satisfy the prespecified type I and II error rates (*αβ*). Simon proposed two criteria to choose an appropriate design among such acceptable designs. The first one minimises the expected sample size under the null hypothesis and is referred to as the ’optimal’ design. The second one minimizes the maximal sample size *n*_*t*_ = *n*_1_ + *n*_2_and is referred to as the ’minimax’ design. Jung *et al.*[18] further proposed a graphical method to search for alternative compromises between Simon’s optimal and minimax designs. For simplicity, we will however here concentrate on the two original Simon’s designs, although all following results may apply to any two-stage design where no early stopping for efficacy is possible.

We suppose here that the sample size of the trial corresponds to the planned *n*_1_ and *n*_2_, and that the stopping rules have been respected at the end of the first stage. Then, as *X*_1_ and *X*_2_are both sums of independent Bernoulli trials, they follow a Binomial distribution of parameters (*n*_1_, *Π*) and (*n*_2_, *Π*), respectively. Let us denote *M* the stopping stage, *S* the total number of response observed at the end of the trial (*S* = *X*_1_ if *M* = 1 and *S* = *X*_*t*_ if *M* = 2), and *N* the total sample size of the trial (*N* = *n*_1_ if *M* = 1 and *N* = *n*_*t*_ if *M* = 2). Jung *et al.*[10] showed that (*MS*) is a complete and sufficient statistic for *Π*, and that the probability mass function of (*MS*) was given by

(1) fΠ(m,s)=n1sΠs(1−Π)n1−sifm=1∑x1=(r1+1)∨(s−n2)s∧n1n1x1n2s−x1Πs(1−Π)nt−sifm=2

for *s* = 1,…,*r*_1_ if *m* = 1 and *s* = *r*_1_ + 1,…,*n*_*t*_ if *m* = 2, and where a∧b=min(a,b) and a∨b=max(a,b).

### Inference following a two-stage design

#### Point estimate

Although the primary goal of phase II trials is decision making rather than inference, obtaining an estimate of the true response rate is often of interest, particularly when the trial was deemed successful and the new drug accepted for further evaluation in phase III trials [7].

The maximum likelihood estimator (MLE) is simply the sample proportion

(2) $${\hat{\Pi }}_{m}=\frac{S}{N}$$

Due to the sequential nature of the trial, the MLE is biased. Actually, in Simon’s design, when extreme small values of *X*_1_ are observed at the first stage, the trial is terminated without a chance to correct the downward bias, leading to a negatively biased MLE. More precisely, the bias is given by

(3) $$b(\Pi )=\frac{1}{n_{1}}\overset{r_{1}}{\underset{x=0}{\sum }}\mathrm{xf}(1,x)+\frac{1}{n_{t}}\overset{n_{t}}{\underset{x=r_{1}+1}{\sum }}\mathrm{xf}(2,x)-\mathrm{\Pi .}$$

Building on prior work of Whitehead [19], Chang *et al.*[9] proposed a bias-adjusted estimator ${\hat{\Pi }}_{w}$ as the numerical solution of

(4) $${\hat{\Pi }}_{w}={\hat{\Pi }}_{m}-b({\hat{\Pi }}_{w}).$$

Guo and Liu [7] proposed a simplified estimator motivated by the same bias substraction idea, but much simpler to obtain numerically by evaluating the bias at the MLE:

(5) $${\hat{\Pi }}_{g}={\hat{\Pi }}_{m}-b({\hat{\Pi }}_{m}).$$

Noting that *X*_1_/*n*_1_ is unbiased for *Π*, an unbiased estimator of *Π* can be obtained by the Rao–Blackwell theorem as the conditional expectation of *X*_1_/*n*_1_ given (*m,s*), where (*m,s*) is the value of (*M,S*) observed in the trial. This estimator was first considered by Chang *et al.*[9] and further studied by Jung *et al.*[10] who showed this estimator was the uniformly minimum variance unbiased estimator (UMVUE). In the case of Simon’s two-stage design, it is given by

(6) Π^u=Sn1ifm=1∑x1=(r1+1)∨(S−n2)S∧n1n1−1x1−1n2S−x1∑x1=(r1+1)∨(S−n2)S∧n1n1x1n2S−x1ifm=2

A median unbiased estimator may be considered as the value of *Π* such that the corresponding *p*-value would be 0.5 (see next section). It was used by Koyama and Chen [11] when *n*_2_ is different from its prespecified value, and will thus be denoted by ${\hat{\Pi }}_{k}$, although they used ${\hat{\Pi }}_{w}$ in their article when *n*_2_ was as planned.

Another approach was used by Tsai *et al.*[12], who restricted their analysis to cases where the trial proceeded to the second stage. In these cases, they derived a (conditional) maximum likelihood estimator of *Π* accounting for the truncated distribution of *X*_1_ (which must be at least *r*_1_ + 1). This conditional estimator will be denoted by ${\hat{\Pi }}_{c}$. To compare all estimators on a fair basis, we assumed that when the trial stopped at the first stage, an unconditional MLE was used. A conditional distribution given *X*_1_≤*r*_1_ may also be derived, but it makes little sense in cases where *r*_1_ is small, in particular when *r*_1_ is 0 or 1, which is the case for optimal and minimax designs for *Π*_0_ = 0.05 and *Π*_1_ = 0.2 or *Π*_1_ = 0.25 with *α*=0.05 and *β*=0.1, for instance. We thus preferred not to consider conditional inference for early trial termination.

Relating to the work of Tsai *et al.*[12], Li recently proposed an MSE-reduced estimator of *Π* as a weighted mean of the naive estimator and ${\hat{\Pi }}_{c}$[14]. This estimator showed slightly higher bias than ${\hat{\Pi }}_{c}$, with a slightly lower MSE, but no clear advantage. It was thus not further considered here.

For inference conditional on proceeding to the second stage, the uniformly minimum variance conditionally unbiased estimator (UMVCUE) can also be obtained, as proposed by Pepe *et al.* who proposed it and studied its properties [13]. Noting that *X*_2_/*n*_2_is unaffected by the early stopping option and thus conditionally unbiased for *Π*, the UMVCUE is obtained similarly to the UMVUE as the conditional expectation of *X*_2_/*n*_2_ given (*m* = 2,*s*). It will be denoted by ${\hat{\Pi }}_{p}$. To provide an estimate when the trial stops at the first stage, several choices are possible, and we decided to use the first stage sample proportion *X*_1_/*n*_1_, which is equal to the UMVUE in this case. For Simon’s design, the UMVCUE can thus be obtained by

(7) Π^p=Sn1ifm=1∑x1=(r1+1)∨(S−n2)S∧n1n1x1n2−1S−x1−1∑x1=(r1+1)∨(S−n2)S∧n1n1x1n2S−x1ifm=2

Numerical studies in various settings showed that the biased-corrected estimators ${\hat{\Pi }}_{w}$ and ${\hat{\Pi }}_{g}$ had often similar performance in terms of bias and mean squared error (MSE), with much smaller bias and slightly higher MSE than the MLE. As compared to the UMVUE, the MLE and the bias-corrected estimators have been shown to have smaller MSE in many situations, but not always [7,10]. Other estimators were not extensively compared to each other or to the previous ones, in particular in the setting of conditional inference or when the actual sample size differes from the preplanned one. Determining in which situation one estimator would be preferable thus remains unclear.

#### *P*-value

Once (*ms*) is observed, the decision rules using critical thresholds *r*_1_and *r*_*t*_ are sufficient to conclude at the rejection of the null hypothesis or not. It remains however common practice to compute a *p*-value at the end of the trial [11]. The first idea that can still be found in many applications is to compute the *p*-value as if the number of responders followed a binomial distribution of parameters (*nΠ*_0_). This yields the naive *p*-value *p*_*n*_

(8) $$\begin{array}{c} p_{n}=\left\{\begin{array}{ll} \underset{\Pi_{0}}{\mathrm{Pr}}(X_{1}\geq s) & \text{if}m=1\\ \overset{n_{1}}{\underset{x_{1}=0}{\sum }}\underset{\Pi_{0}}{\mathrm{Pr}}(X_{1}=x_{1})\underset{\Pi_{0}}{\mathrm{Pr}}(X_{2}\geq s-x_{1}) & \text{if}m=2 \end{array}\right) \end{array}$$

The assumption on the distribution of *S* is true if *m* = 1, but obviously wrong if *m* = 2. This is exemplified on equation (7) by the summation on impossible sample paths where *X*_1_<*r*_1_ and *X*_2_ = *s*−*X*_1_.

It is therefore necessary to use the proper distribution of observed data to compute a *p*-value. The *p*-value is the probability under the null hypothesis to obtain a result at least as extreme as the one observed. Owing to the multistage procedure, several orderings, i.e. several definitions of ”at least as extreme”, may however be considered even if the proper distribution is used [20]. For instance, assume a design with *n*_1_=24, *n*_2_=39, *r*_1_=8 and *r*_*t*_=24 (optimal design for *Π*_0_=0.30, *Π*_1_=0.50, *α*=0.05 and *β*=0.10). One may consider that obtaining 18 responders out of 63 patients after proceeding to the second stage is less extreme than obtaining 7 responders out of 24 patients and stopping at the first stage, because 18/63=0.286 is less than 7/24=0.292. This corresponds to MLE ordering [20,21]. Conversely, one may also use stage-wise ordering, and consider that 18/63 is a more extreme result than 7/24 because it was observed after proceeding to the second stage instead of stopping at the first stage. Indeed, to proceed to the second stage the number of responders in the first stage was at least 9. This is the ordering recommended in Jennison and Turnbull in the general case of sequential clinical trials [20, chapter 18.4, p 180], and the one they use to compute exact confidence bounds for *Π*[22].

The *p*-value based on MLE ordering is

(9) $$p_{m}=\underset{\left\{(i,j):{\hat{\Pi }}_{m}(i,j)\geq {\hat{\Pi }}_{m}(m,s)\right\}}{\sum }f_{\Pi_{0}}(i,j)$$

The bias-corrected estimators have the same ordering as the MLE [23]. They thus result in exactly the same *p*-value.

Jung *et al.*[10] showed that UMVUE ordering is equivalent to stage-wise ordering and later defined a *p*-value based on this ordering as [23]

(10) $$p_{s}=\left\{\begin{array}{ll} 1-\underset{\left\{(i,j):{\hat{\Pi }}_{u}(i,j)<{\hat{\Pi }}_{u}(m,s)\right\}}{\sum }f_{\Pi_{0}}(i,j) & \text{if}m=1\\ \underset{\left\{(i,j):{\hat{\Pi }}_{u}(i,j)\geq {\hat{\Pi }}_{u}(m,s)\right\}}{\sum }f_{\Pi_{0}}(i,j) & \text{if}m=2 \end{array}\right)$$

It can be rewritten as

(11) $$\begin{array}{c} p_{s}=\left\{\begin{array}{ll} \underset{\Pi_{0}}{\mathrm{Pr}}(X_{1}\geq s) & \text{if}m=1\\ \overset{n_{1}}{\underset{x_{1}=r_{1}+1}{\sum }}\underset{\Pi_{0}}{\mathrm{Pr}}(X_{1}=x_{1})\underset{\Pi_{0}}{\mathrm{Pr}}(X_{2}\geq s-x_{1}) & \text{if}m=2 \end{array}\right) \end{array}$$

which is equivalent to the *p*-value given by Koyama-Chen for designs where attained *n*_2_ is as planned [11].

When estimation is performed conditional on proceeding to the second stage, a conditional *p*-value can also be proposed. Let us denote *f*_*Π*_(*s*|*m* = 2) the probability mass function of *S* conditional on *m* = 2,

(12) $$f_{\Pi }(s|m=2)=\frac{f_{\Pi }(m,s)}{\overset{n_{1}}{\underset{x_{1}=r_{1}+1}{\sum }}\Pi^{x_{1}}{\left(1-\Pi \right)}^{n_{1}-x_{1}}},$$

where *f*_*Π*_(*m*,*s*) is given in (1). When the trial proceeds to the second stage, the conditional *p*-value *p*_*c*_ is computed by

(13) $$p_{c}=\overset{n_{t}}{\underset{i=s}{\sum }}f_{\Pi_{0}}(i|m=2).$$

If the trial is stopped at the first stage, *p*_*c*_ can simply be computed by $\underset{\Pi_{0}}{\mathrm{Pr}}(X_{1}\geq s)$ and is thus equal to *p*_*s*_.

#### Confidence interval

Beside point estimates, confidence intervals are often reported in phase II trials. Despite the one-sided nature of Simon’s design, it is not uncommon to report two-sided (1−2*α*) confidence intervals rather than left (1−*α*) one-sided confidence intervals. We will thus make this choice although both approaches are consistent with the one-sided test performed at level *α*. Note however that in many applications, two-sided 95% confidence intervals are reported, whatever the choice on the (one-sided) *α*level.

The first basic idea is to use Clopper–Pearson [24] exact confidence interval ignoring the group sequential nature of the trial. We refer to this approach as the naive exact confidence interval in the sequel. Another solution is to use the Clopper–Pearson definition of an exact confidence interval using the appropriate distribution of (*MS*) [20]. This defines the exact equal tail (1−2*α*) confidence interval as (*Π*_1_*Π*_2_), where *Π*_1_ and *Π*_2_ are the numerical solutions of

(14) $${\text{Pr}}_{\Pi_{1}}\left[{\hat{\Pi }}_{u}(M,S)\geq {\hat{\Pi }}_{u}(m,s)\right]=\alpha$$

and

(15) $${\text{Pr}}_{\Pi_{2}}\left[{\hat{\Pi }}_{u}(M,S)\leq {\hat{\Pi }}_{u}(m,s)\right]=\mathrm{\alpha .}$$

The existence of this interval relies on the stochastic ordering of the distribution of (*M,S*) with respect to *Π*[10]. It is the same as the confidence interval used in several other works [11,22]. As it uses the UMVUE or stage-wise ordering, we refer to it as the exact stage-wise confidence interval. Using MLE ordering instead of stage-wise ordering does not result in the same property of stochastic ordering [10]. It was therefore not further considered.

In the simple setting of a single binomial proportion, the Clopper–Pearson confidence interval is known to be conservative [25]. Actually, the actual confidence level is bounded below by (1−2*α*) [26]. To correct for this conservative nature, it has been suggested to use so-called mid-*p* confidence intervals [27]. We thus extended the stage-wise ordering confidence intervals with a mid-*p* approach as ($\Pi_{1}^{\prime }\text{,}\Pi_{2}^{\prime }$), where $\Pi_{1}^{\prime }$ and $\Pi_{2}^{\prime }$ are the numerical solutions of

(16) $$\begin{array}{c} {\text{Pr}}_{\Pi_{1}^{\prime }}\left[{\hat{\Pi }}_{u}(M,S)>{\hat{\Pi }}_{u}(m,s)\right]\;+\;\frac{1}{2}{\text{Pr}}_{\Pi_{1}^{\prime }}\left[{\hat{\Pi }}_{u}(M,S)\;=\;{\hat{\Pi }}_{u}(m,s)\right]\;=\;\alpha \end{array}$$

and

(17) $$\begin{array}{c} {\text{Pr}}_{\Pi_{2}^{\prime }}\left[{\hat{\Pi }}_{u}(M,S)\;<{\hat{\Pi }}_{u}(m,s)\right]\;+\;\frac{1}{2}{\text{Pr}}_{\Pi_{2}^{\prime }}\left[{\hat{\Pi }}_{u}(M,S)\;=\;{\hat{\Pi }}_{u}(m,s)\right]\;=\;\mathrm{\alpha .} \end{array}$$

Tsai *et al.*[12] considered several other intervals, both asymptotic and exact, but focusing on cases were the trial proceeds to the second stage, and using conditional inference as stated earlier. Asymptotic confidence intervals considered were the Wald and score intervals, both with or without continuity correction, and based on the conditional MLE given the trial proceeds to a second stage (referred as MLE in their article). Exact confidence intervals were Clopper–Pearson as explained above, but based on the conditional distribution of (*M,S*) given *m* = 2 (equation 10), and Sterne exact interval, modified to obtain an interval when the original method produces disjoint intervals as a confidence region. They concluded upon recommendation of score confidence intervals with continuity correction. Only the latter and Clopper–Pearson intervals will thus be considered here, and referred as the conditional score and conditional exact confidence intervals. Moreover, we proposed a mid-*p* confidence interval using the conditional distribution of (*M,S*) given *m* = 2. It is referred as the conditional mid-*p* confidence interval. Pepe *et al.* used parametric and nonparametric bootstrap confidence intervals for the UMVCUE in their article [13]. They showed that both methods yielded coverage probabilities reasonably close to the nominal level, but lower for the parametric bootstrap than for the nonparametric bootstrap. However, these methods do not provide correct confidence intervals in some situations, for instance when *X*_2_=0 or *s* = *n*_*t*_. They were thus not considered here.

### Extended or shortened trial

It is not uncommon that the actual sample size of a phase II trial would be different from the planned sample size [11,15]. This may be due to differences between anticipated and actual accrual and drop out rates, for instance. For a two stage design, current practice often relies on ignoring the over- or underaccrual or in re-computing the decision boundaries as if the attained sample size had been planned in a single-stage design, which leads to bias and possible inflation of the type I error rate. Koyama and Chen [11] recently proposed a method to calculate a new critical value for the second stage analysis assuming dropouts and overrun would be totally non-informative. In this case, the interim analysis can always be performed on the preplanned *n*_1_subjects, and the difference in sample size only concerns the second stage sample size. They also proposed a method for inference at the end of the trial, thus providing a point estimate, a confidence interval and a *p*-value.

Assume $n_{2}^{\prime }$=*n*_2_ + *Δn*_2_ patients are accrued at the second stage instead of the preplanned *n*_2_, and that $X_{2}^{\prime }$ success are then observed, where $X_{2}^{\prime }$ follows a binomial distribution of parameters ($n_{2}^{\prime ,\Pi }$). Briefly, the method proposed consists in defining a new critical value for the second stage as the one leading to the same decision as when comparing the conditional *p*-value of the second stage Pr${}_{\Pi_{0}}(X_{2}^{\prime }\geq x_{2}^{\prime }|X_{1}=x_{1})$ to the conditional type I error rate given *X*_1_ = *X*_1_ in the original design with *n*_2_ patients at the second stage. The new conditional type I error rate is thus lower or equal to the original conditional type I error rate, allowing to control the unconditional type I error rate.

They also proposed to compute the unconditional *p*-value as

(18) $$p_{k}=\overset{n_{1}}{\underset{x_{1}=r_{1}+1}{\sum }}{\text{Pr}}_{\Pi_{0}}\left(X_{1}=x_{1}\right)A(x_{1},n_{2},\Pi^{*}),$$

where A(x1,n2,Π)=∑x2=rt−x1+1n2n2x2Πx2(1−Π)(n2−x2) is the conditional power function at the second stage, and ^*Π*∗^is the solution of $A(x_{1},n_{2},\Pi^{*})=\underset{\Pi_{0}}{\mathrm{Pr}}(X_{2}^{\prime }\geq x_{2}^{\prime }|X_{1}=x_{1})$. Solving for ^*Π*∗^ allows to extend the conditional power to all potential values of *X*_1_, whereas only one particular value (*X*_1_) was observed. The use of the conditional power function $A(x_{1},n_{2},\Pi^{*})$ allows ordering different sample paths with different *X*_1_ and the actual sample size for stage 2 $n_{2}^{\prime }$ by comparing the ^*Π*∗^, smaller ^*Π*∗^ indicating stronger evidence against the null hypothesis. This ordering is coherent with the hypothesis testing strategy they proposed, based on a new critical value to control the conditional type I error. In that respect, the *p*-value _*pk*_ is lower than *α* if and only if the null hypothesis is rejected.

Koyama and Chen proposed the estimator ${\hat{\Pi }}_{k}$ as the value of *Π*_0_ yielding a *p*-value *p*_*k*_=0.5, and a two-sided Clopper–Pearson-like confidence interval based on *p*_*k*_. The definition of *p*_*k*_ by equation 11 should allow to control the overall type I error rate, but the properties of the test, estimator and confidence interval have not been thoroughly studied.

Although Koyama and Chen used a biased-corrected estimator when the second stage sample size was as planned, we denoted ${\hat{\Pi }}_{k}$ the median estimator presented above also in the case where *n*_2_ patients are accrued at the second stage.

### Numerical study

To examine the properties of the different methods, numerical studies were conducted. Several design scenarios were considered, that covered a range of possible phase II trials in oncology. To help determining these scenarios, a limited literature search of phase II cancer trials using Simon’s design over the last years was performed. As this study was informal and arbitrarily limited to some journals, no results are reported. Twelve design scenarios where thus considered, with response rates under the null hypothesis of 0.05, 0.1, 0.2, 0.3, 0.4 and 0.5. Trials with higher values of *Π*_0_were considered as pretty rare, and therefore not considered. For each value of *Π*_0_, two differences in response rate between the null and alternative hypotheses were considered, namely 0.15 and 0.2. In all cases, the type I error rate *α* was set to 0.05 and the type II error rate *β* to 0.10 (90% power). Then, for each combination of design parameters, a choice between Simon’s optimal and minimax design was made on a case by case basis, according to the expected total sample size of the trial and the probability of early termination under H_0_ and H_1_.

For each design scenario considered, the probability of all possible outcomes (*M*,*S*) was computed using equation (1) for a range of values of the response rate *Π* varying from *Π*_0_ to *Π*_0_ + 0.20 (thus *Π*_1_ when *δ* was 0.20 and slightly more than *Π*_1_when *δ* was 0.15). For each possible outcome, the resulting estimators, *p*-values and confidence intervals were also computed. As the probability of each outcome was the probability distribution of the estimators, *p*-values and confidence intervals, the bias and root mean square error (RMSE) of estimators, the probability of rejection of the tests based on the *p*-values and coverage probability of the confidence intervals could be derived.

To investigate the impact of accrual of some more or some fewer patients at the second stage as compared to the planned *n*_2_ value, trials where the second stage sample size was decreased by 1 or 2 or increased by 1, 2 or 5 were considered. These settings were not symmetrical because it was felt that overaccrual would be more frequent, because of the time delay to close a trial and because investigators would more likely want to protect the trial from patients exclusion and thus easily accrue more patients. Main analysis was unconditional: i.e. performance of the different methods was averaged over all possible outcomes. As some methods were more specifically developed to correct the analysis of the second stage results only, analysis restricted to cases where the trial proceeded to a second stage was also performed, and referred as conditional analysis.

To keep results simple and because the main findings were close to one scenario or another, only the results of six of the twelve scenarios are presented in detail. Additionally, these detailed results are only presented for situations where the second stage sample size was as planned. For situations where the second stage sample size was different from planned, the tables present results averaged over the different scenarios and the different values of *Δn*_2_ (simple arithmetic average without any weighting). However, the description of results encompassed the whole range of data obtained and not only the results presented in the tables. Particular cases where results were representative or different from the overall message were then isolated.

All computations were performed using R 2.13.2 statistical software [28].

## Results

### Trial accrual as planned

**Figure 1:**
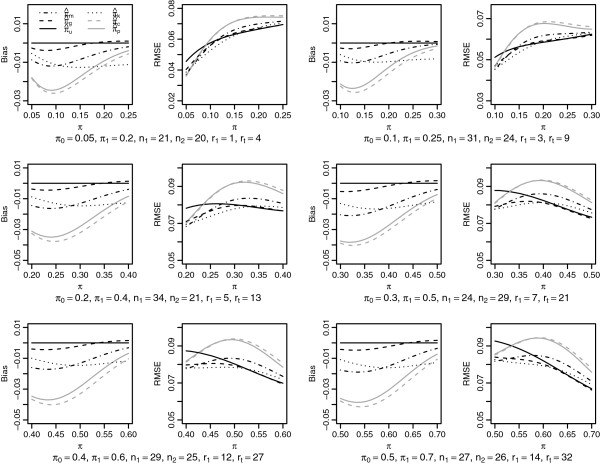
Performance of the estimators: bias and root mean squared error (RMSE).

Results displayed in Figure 1 show that the UMVUE ${\hat{\Pi }}_{u}$ has no marginal bias as expected, the bias corrected estimator ${\hat{\Pi }}_{g}$ is almost unbiased, and if the median unbiased estimator ${\hat{\Pi }}_{k}$ and the MLE ${\hat{\Pi }}_{m}$ are biased, the bias remains limited, lower that 2% for the considered scenarios. In terms of RMSE, ${\hat{\Pi }}_{g}$ perfoms better than ${\hat{\Pi }}_{u}$ for values of *Π* closer to *Π*_0_ than to *Π*_1_, while the RMSE of both estimators become similar when *Π*approaches *Π*_1_. As already noted in the illustrative examples of Guo and Liu [7], the MLE has the smallest RMSE under H_0_. The median estimator also perfoms well in terms of RMSE, and even exhibits the smallest one for values of *Π* near *Π*_0_. The conditional estimators have similar properties to each other, with much higher negative bias than the MLE, especially for values of *Π* close to *Π*_0_. They had also higher or equal RMSE than the MLE.

**Figure 2:**
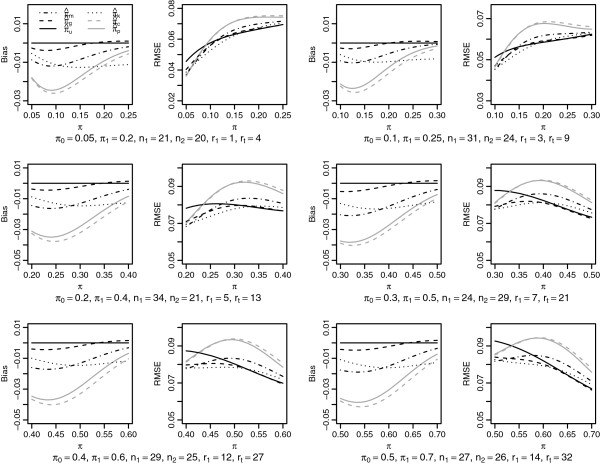
**Performance of the tests based on *p* -values and the two-sided 90% confidence intervals: probability of rejection and coverage probability.** The line denoted by ’Design’ presents the probability of rejection according to the trial’s design i.e. when *X*_*t*_>*r*_*t*_.

In terms of statistical testing, the test sizes represented on Figure 2 when *Π* = *Π*_0_ show that the naive binomial test and the test based on the conditional distribution are not adequate, these tests being too conservative in several settings. The test based on stage-wise ordering leads to the correct decision, with the same probability of rejection as given by design. In our numerical settings, the test based on MLE ordering had similar characteristics as the test based on stage-wise ordering. Actually, both only differ for a limited range of possible (*M*,*S*) outcomes, which has no impact in terms of test conclusion in the situations covered by the numerical study, although the nominal *p*-values may be different.

Coverage probabilities of the 90% confidence intervals are presented in the right sub-panel of Figure 2 for each design scenario. Overall, the properties of all methods but the mid-*p* approach where disappointing, in particular for small values of *Π*_0_ such as 0.05 for instance. The mid-*p* confidence interval had coverage probabilities closer to the nominal level than the other approaches in almost all situations. It was conservative under H_0_ for smaller values of *Π*_0_, but the coverage probability fluctuated around 90% when *Π*_0_ was 0.20 or more, within a margin of −1*%*to + 2*%* only. On the contrary, the exact (stage-wise ordering) confidence intervals had always a coverage probability above 90%, but often 2 to 3% above, and even between 7 and 8% above for smaller sample size trials. The conservative nature of Clopper–Pearson approach has already been reported, and the performance observed here for such intervals was however not clearly worse as that reported for so-called exact confidence intervals in a one sample (one-stage) setting [25]. Note that the phenomenon of oscillations in coverage probability according to *Π* appearing on the graphs is known, and caused by the lattice structure of the binomial distribution [29]. The confidence intervals based on the conditional score with continuity correction which exhibited better conditional performance in the work by Tsai *et al.*[12] and the conditional mid-*p* confidence interval had close performance, but for *Π* departing from *Π*_0_, their coverage probabilities were lower than the nominal level in this unconditional setting. This occurred less frequently and less dramatically for the conditional exact confidence interval, which however had a coverage probability clearly above its nominal level for *Π* close to *Π*_0_, especially for small values of *Π*_0_.

### Extended or shortened trial

Results obtained when the second stage sample size was modified are presented in Tables 1 (average over all scenarios) and 2 for some of the situations. When the actual number of patients accrued was a little smaller or larger than planned, the UMVUE still yielded an unbiased estimator of the response rate. This was expected as the UMVUE is obtained as the conditional expectation of the first stage proportion given (*M,S*), without using any information on the decision boundaries at the second stage. If more or less patients are accrued in stage 2, this implies modifying this boundary to control for the type I error rate, but it has no impact on estimation. All other estimators were biased. In particular, Koyama–Chen method, aiming at correcting for increased or decreased sample size at the second stage also yielded an unconditionnally biased estimator, with bias and RMSE even superior to Guo’s corrected estimator. Both had however smaller RMSE than the UMVUE in most cases. The UMVCUE estimator and the conditional estimator ${\hat{\Pi }}_{c}$ had larger bias than the others under H_0_, but their bias under *H*_1_ was similar to the one of Koyama–Chen estimator, with even lower RMSE for the UMVCUE.

**Table 1:** Performance of the different methods when second stage sample size was different from planned: average over the different design scenarios and differences between the planned and attained second stage sample size

Property	Method	*Π=Π*_0_	*Π=Π*_0_* + δ*
Bias	${\hat{\Pi }}_{m}$	−0.015	−0.005
	${\hat{\Pi }}_{g}$	−0.004	0.001
	${\hat{\Pi }}_{u}$	0.000	0.000
	${\hat{\Pi }}_{c}$	−0.029	−0.012
	${\hat{\Pi }}_{p}$	−0.028	−0.009
	${\hat{\Pi }}_{k}$	−0.009	−0.012
RMSE	${\hat{\Pi }}_{m}$	0.060	0.071
	${\hat{\Pi }}_{g}$	0.063	0.067
	${\hat{\Pi }}_{u}$	0.071	0.067
	${\hat{\Pi }}_{c}$	0.061	0.076
	${\hat{\Pi }}_{p}$	0.062	0.064
	${\hat{\Pi }}_{k}$	0.062	0.070
Rejection probability	*p*_*n*_	0.033	0.882
	_*pm*_	0.036	0.887
	_*pu*_	0.036	0.887
	*p*_*c*_	0.012	0.800
	_*pk*_	0.035	0.885
Coverage probability	Naive exact	0.940	0.916
	Stage-wise	0.937	0.933
	Mid-*p*	0.916	0.895
	Conditional exact	0.952	0.906
	Conditional score	0.935	0.851
	Conditional mid-*p*	0.936	0.860
	Koyama–Chen	0.937	0.931

**Table 2:** Performance of the estimators when second stage sample size is modified by *Δn*_2_: bias and root mean squared error in selected situations

			***Δn***_**2**_***=−*2**		***Δn***_**2**_***=−*1**		***Δn***_**2**_***= + *1**		***Δn***_**2**_***= + *2**										***Δn***_**2**_***= + *5**	
**Settings**	**Estimator**		**Bias**	**RMSE**	**Bias**	**RMSE**	**Bias**	**RMSE**	**Bias**	**RMSE**	**Bias**	**RMSE**								
Optimal design with *Π*_0_ = 0.05, *Π*_1_ = 0.2: *n*_1_=21, *n*_2_=20, *r*_1_=1, *r*_*t*_=4											
*Π* = *Π*_0_	${\hat{\Pi }}_{m}$		-0.008	0.038	-0.009	0.037	-0.009	0.037	-0.009	0.037	-0.010	0.036
	${\hat{\Pi }}_{g}$		-0.002	0.041	-0.003	0.041	-0.003	0.040	-0.003	0.040	-0.003	0.040
	${\hat{\Pi }}_{u}$		0.000	0.046	0.000	0.046	0.000	0.046	0.000	0.045	0.000	0.045
	${\hat{\Pi }}_{c}$		-0.018	0.036	-0.018	0.036	-0.018	0.036	-0.018	0.036	-0.018	0.035
	${\hat{\Pi }}_{p}$		-0.018	0.037	-0.018	0.037	-0.018	0.036	-0.018	0.036	-0.018	0.035
	${\hat{\Pi }}_{k}$		-0.006	0.039	-0.006	0.039	-0.006	0.038	-0.006	0.038	-0.006	0.038
*Π* = *Π*_1_	${\hat{\Pi }}_{m}$		-0.004	0.071	-0.004	0.071	-0.005	0.069	-0.005	0.069	-0.005	0.067
	${\hat{\Pi }}_{g}$		0.001	0.068	0.001	0.068	0.001	0.066	0.001	0.066	0.001	0.064
	${\hat{\Pi }}_{u}$		0.000	0.068	0.000	0.067	0.000	0.066	0.000	0.065	0.000	0.064
	${\hat{\Pi }}_{c}$		-0.012	0.077	-0.012	0.076	-0.011	0.074	-0.011	0.073	-0.011	0.071
	${\hat{\Pi }}_{p}$		-0.009	0.076	-0.009	0.075	-0.009	0.074	-0.009	0.073	-0.009	0.071
	${\hat{\Pi }}_{k}$		-0.012	0.071	-0.013	0.070	-0.013	0.069	-0.013	0.068	-0.013	0.067
Minimax design with *Π*_0_=0.4, *Π*_1_=0.6: *n*_1_=29, *n*_2_=25, *r*_1_=12, *r*_*t*_=27												
*Π* = *Π*_0_	${\hat{\Pi }}_{m}$		-0.015	0.078	-0.016	0.078	-0.016	0.077	-0.017	0.077	-0.018	0.076
	${\hat{\Pi }}_{g}$		-0.004	0.080	-0.004	0.080	-0.004	0.080	-0.004	0.079	-0.004	0.079
	${\hat{\Pi }}_{u}$		0.000	0.087	0.000	0.087	0.000	0.087	0.000	0.087	0.000	0.087
	${\hat{\Pi }}_{c}$		-0.037	0.082	-0.037	0.082	-0.036	0.081	-0.036	0.080	-0.036	0.079
	${\hat{\Pi }}_{p}$		-0.035	0.083	-0.035	0.082	-0.035	0.081	-0.035	0.081	-0.035	0.080
	${\hat{\Pi }}_{k}$		-0.010	0.079	-0.010	0.078	-0.010	0.078	-0.010	0.078	-0.010	0.078
*Π* = *Π*_1_	${\hat{\Pi }}_{m}$		-0.003	0.074	-0.003	0.074	-0.003	0.073	-0.003	0.073	-0.003	0.071
	${\hat{\Pi }}_{g}$		0.001	0.070	0.001	0.070	0.002	0.069	0.002	0.068	0.002	0.067
	${\hat{\Pi }}_{u}$		0.000	0.071	0.000	0.070	0.000	0.069	0.000	0.069	0.000	0.068
	${\hat{\Pi }}_{c}$		-0.011	0.082	-0.011	0.081	-0.010	0.080	-0.010	0.079	-0.010	0.077
	${\hat{\Pi }}_{p}$		-0.007	0.080	-0.007	0.079	-0.007	0.078	-0.007	0.077	-0.007	0.076
	${\hat{\Pi }}_{k}$		-0.012	0.073	-0.012	0.072	-0.011	0.071	-0.011	0.071	-0.011	0.070

In terms of hypothesis testing and *p*-values, all methods except the conditional test yielded very close results, with no increase of the type I error rate in the situations studied. Actually, the possible values of (*M*,*S*) where these methods disagreed in terms of rejection of the null hypothesis had very small probabilities in general, thus almost no impact on test size or power. In several situations, there were even no values of (*M*,*S*) for which the methods disagreed. On the contrary, the test based on the conditional *p*-value had a probability of rejection markedly smaller than other methods, with both a type I error rate and a power clearly under their nominal value.

The mid-*p* confidence intervals had again coverage probabilities closer to the nominal 90% level than the other methods, in particular than the Koyama–Chen method which was corrected for sample size modifications. Over all 120 situations covered, the Koyama–Chen confidence intervals were rather conservative but always preserved the nominal confidence level, with coverage probabilities ranging from 90.0% to 98.5%, with an average of 93.4%. On the contrary, coverage probabilities ranged from 85.7% to 96.5% for the mid-*p* confidence intervals, with an average of 90.1%. Coverage probabilities under the nominal level were more frequent under H_1_ than under H_0_ and for higher values of the probability of response *Π*.

### Analysis conditional on proceeding to stage 2

**Figure 3:**
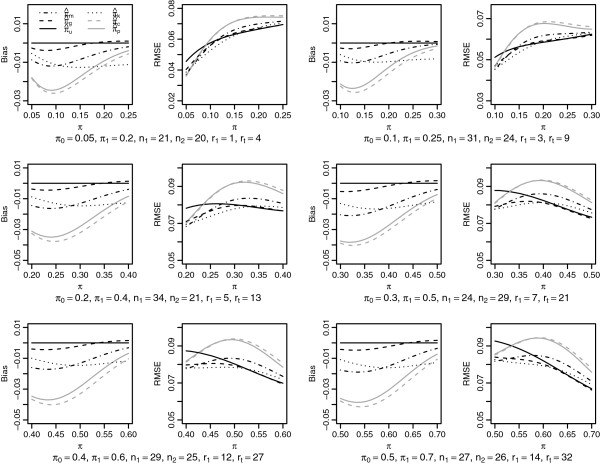
Performance of the estimators for conditional inference: bias and root mean squared error (RMSE).

When analysis was restricted to the trials proceeding to the second stage, the performance of the estimators was different from previously (Figure 3). The UMVCUE of Pepe *et al.* was unbiased, whereas the conditional estimator of Tsai *et al.* had very small negative bias. All other estimators were positively biased, with marked bias under the null hypothesis that decreased when the true response rate increased towards the alternative hypothesis. Overall, the MLE estimator had less bias than Guo’s corrected estimator and the UMVUE. Interestingly, the Koyama–Chen estimator was even slightly negatively biased for *Π* close to *Π*_1_ or above, with a bias of the same magnitude than the bias of the conditional estimator ${\hat{\Pi }}_{c}$ under H_1_.

In terms of RMSE, the conditional estimators ${\hat{\Pi }}_{c}$ and ${\hat{\Pi }}_{p}$ had close performance, with negligible differences in favor of ${\hat{\Pi }}_{c}$ under H_0_ and of ${\hat{\Pi }}_{p}$ under H_1_. Despite their bias, all unconditional estimators except the UMVUE had generally lower RMSE than the conditional estimators. With biases as high as 4% for response rate of 5% or as 8% for a response rate of 20%, these estimators cannot be recommended for conditional inference, however.

**Figure 4:**
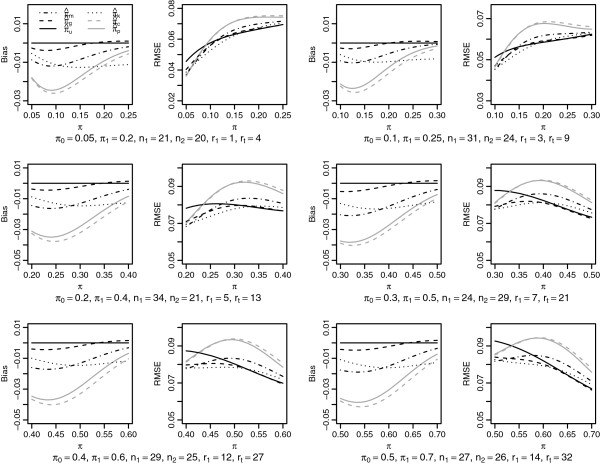
**Performance of the tests based on *p* -values and the two-sided 90% confidence intervals for conditional inference: probability of rejection and coverage probability.**

Conditional inference was also the only one preserving the conditional type I error, but the test could be rather conservative in some situations (Figure 4). As a consequence, the power conditional on proceeding to the second stage could be lower than 90% in some cases. As described in Tsai *et al.*[12], the conditional score performed better than the conditional exact confidence interval. The conditional mid-*p* confidence interval had coverage probabilities very close to the conditional score interval.

When the sample size at the second stage *n*_2_ was different from its planned value, the conditional estimators achieved similar bias reduction as when *n*_2_ was as planned (Table 3). In particular, the UMVCUE was virtually unbiased, at least in all designs scenarios considered here. The test based on the conditional *p*-value *p*_*c*_also allowed to control the conditional type I error. The coverage probabilities of conditional score and conditional mid-*p* confidence intervals tended to be higher under H_0_ than under H_1_, and closer to their nominal value under *H*_1_, whereas the reverse was observed for other methods. As compared to the conditional estimator, Koyama–Chen estimator had similar bias and lower RMSE under H_1_, but much higher bias under H_0_. It should however be noted that this estimator is constructed as a median and not a mean estimator, so that some degree of bias can be expected when estimating the response rate. In terms of hypothesis testing, this method however failed to adequately control the conditional type I error rate and confidence intervals had too high coverage probability in most cases.

**Table 3:** Performance of the different methods for conditional inference when second stage sample size was different from planned: average over the different scenarios

**Property**	**Method**	***Π=Π***_**0**_	***Π=Π***_**0**_*** + δ***
Bias	${\hat{\Pi }}_{m}$	0.038	0.004
	${\hat{\Pi }}_{g}$	0.053	0.010
	${\hat{\Pi }}_{u}$	0.084	0.010
	${\hat{\Pi }}_{c}$	−0.003	−0.002
	${\hat{\Pi }}_{p}$	0.000	0.000
	${\hat{\Pi }}_{k}$	0.057	−0.003
RMSE	${\hat{\Pi }}_{m}$	0.057	0.059
	${\hat{\Pi }}_{g}$	0.068	0.056
	${\hat{\Pi }}_{u}$	0.086	0.054
	${\hat{\Pi }}_{c}$	0.060	0.065
	${\hat{\Pi }}_{p}$	0.061	0.064
	${\hat{\Pi }}_{k}$	0.062	0.057
Rejection probability	*p*_*n*_	0.100	0.931
	_*pm*_	0.110	0.936
	_*pu*_	0.110	0.936
	*p*_*c*_	0.035	0.844
	_*pk*_	0.107	0.933
Coverage probability	Naive exact	0.899	0.939
	Stage-wise	0.890	0.957
	Mid-*p*	0.852	0.941
	Conditional exact	0.939	0.929
	Conditional score	0.910	0.894
	Conditional mid-*p*	0.913	0.903
	Koyama–Chen	0.889	0.956

## Discussion

In terms of estimation, ${\hat{\Pi }}_{g}$ and ${\hat{\Pi }}_{u}$ should be recommended as they perform better than the other estimators, in particular when the true response rate is higher than the one under H_0_, i.e. in cases when estimation is the most important. Although our simulations did not encompass all possible ranges of response rates and treatment effects, they cover a wide range of plausible situations, in which no clear advantage of the bias corrected estimator ${\hat{\Pi }}_{g}$ over the UMVUE ${\hat{\Pi }}_{u}$ could be found.

The choice of a conditional or unconditional inference is clearly overlooked in practical applications. Conditional inference — and conditional bias in particular — has attracted some interest in the setting of group sequential phase III trials, with concerns rather directed at the conditional bias of the estimator of the treatment effect when trials were stopped early for efficacy [30,31]. In the setting of Simon’s two-stage phase II trials, conditional inference would rather be favored when the trial did not stop at the first stage, especially if the trial was deemed succesful at the end [13]. Such aspects of conditional inference have however been rarely discussed to our knowledge [13,32]. Results show that unbiased or almost unbiased estimation can be performed using the UMVCUE [13] or the proper conditional distribution [12], respectively, both with very similar RMSE. In addition, both performed well even when the sample size at the second stage was slightly different from its planned value. To construct an estimator that would be both conditionally and unconditionally unbiased, one could also derive an estimator for trials stopping at the first stage that would use the conditional distribution given *X*_1_≤*r*_1_. In such a case, the estimator would be conditionally unbiased whether the trial was stopped at the first or the second stage, and thus would be unconditionally unbiased. Using a distribution of outcomes conditional on early stopping makes however little sense — if any — when *r*_1_ is small. For instance, if *r*_1_=0, then the only potential outcome in case of early stopping is *X*_1_=0, thus leading to a single possible value for the estimator of *Π*. It is therfore not possible to construct an unbiased estimator of any value of *Π* in this case. We therefore did not further develop this point in the paper. Another solution, however, would be to use a biased-corrected estimator such as Whitehead’s [19] or Guo’s [7] when the trial was stopped early. This has already been evoked by Pepe *et al.*[13], without further investigations.

In this study, we have concentrated on Simon’s design for phase II cancer trials. Other designs or adaptations however exist. In particular, Jovic and Whitehead have recently proposed point estimates, confidence intervals and *p*-values for a modified Simon’s design with early stopping for efficacy [33]. Other extensions of Simon’s design could also have been considered [5,34]. In cases where early stopping for efficacy is possible, the results of the methods proposed by Jovic and Whitehead could have been used. Tsai *et al.* also applied their conditional method to Shuster’s design [34]. Nevertheless, a short look at cancer literature shows that a majority of cancer phase II trials still use Simon’s design.

In practical applications, it may occurr that the actual number of patients recruited would be slightly different from the preplanned value. For instance some patients may be unevaluable for response or they may withdraw their consent during study. On the contrary, some patients may be included in the study before recruitment is formally closed. For these cases, where the decrease or increase of second stage sample size may be considered as non informative, Koyama and Chen proposed inference procedures based on conditional power [11]. They clearly state in their article that the properties of their estimators, *p*-values and confidence intervals need to be further studied. In our numerical settings, it turned out that the UMVUE, which can still be used because it only makes use of boundary decisions at the second stage, performed better than the Koyama–Chen method. The behaviour of both estimators with modified sample size however deserve further investigations. Concerning confidence intervals, the mid-*p* intervals performed better than the so-called exact confidence intervals in most settings for both unconditional and conditional inference. Koyama and Chen however did not consider such an approach, and their confidence intervals rely on Clopper–Pearson method. Using a mid-*p* approach with their modifed *p*-value (equation 11) may also have improved the coverage probabilities of the confidence intervals.

Another interesting field of further research concerns inference in adaptive phase II trials, where the second stage sample size can be adapted according to the first stage results [16,17]. In such cases, the decrease or increase in sample size cannot be considered as non informative anymore, and the method of Koyama and Chen does not apply. New developments are thus needed here.

## Conclusions

For point estimation, the UMVUE ${\hat{\Pi }}_{u}$ was unbiased both when the actual number of patients recruited was equal to or differed from the preplanned value. The bias corrected estimator ${\hat{\Pi }}_{g}$ had negligible bias and slightly lower RMSE than the UMVUE only when the true response rate *Π* was close to its value under the null hypothesis. Both estimators perfomed better than the others and can thus be recommended. In terms of confidence intervals, mid-*p* confidence intervals performed best, as compared to the other exact confidence intervals, whether they ignore the group sequential nature of the trial or not.

When one is more particularly interested on inference conditional on having proceeded to the second stage, the UMVCUE ${\hat{\Pi }}_{p}$ which is unbiased may be recommended. Conditional score or conditional mid-*p* confidence intervals should then be used.

## Competing interests

The authors declare that they have no competing interests.

## Authors’ contributions

RP and KD designed the study, performed all statistical analyses and participated to article writing. Both authors read and approved the final manuscript.

## Pre-publication history

The pre-publication history for this paper can be accessed here:

http://www.biomedcentral.com/1471-2288/12/117/prepub
